# Comparison of Stored and Fresh Injectable Acellular Adipose Matrix in Soft Tissue Reconstruction in a Murine Model

**DOI:** 10.1007/s00266-024-04175-y

**Published:** 2024-06-24

**Authors:** Jaewoo Kim, Vinh Vuong The Tran, Ki Yong Hong, Hak Chang

**Affiliations:** 1 Department of Plastic and Reconstructive Surgery, Seoul National University College of Medicine, Seoul, Republic of Korea; 2 Department of Plastic and Reconstructive Surgery, SMG-SNU Boramae Medical Center, Seoul, Republic of Korea; 3 Hi-Tech Center, Vinmec Healthcare System, Hanoi, Vietnam; 4 Department of Plastic and Reconstructive Surgery, Seoul National University Hospital, Seoul National University College of Medicine, 101 Daehak-ro, Jongno-gu, Seoul, 03080 Republic of Korea

**Keywords:** Acellular adipose matrix, Injectable, Retention rate, Storage period

## Abstract

**Background:**

We previously showed comparable volume effects of injections of acellular adipose matrix (AAM), an adipose tissue-derived extracellular matrix, and conventional fat grafting in a murine model. Thus, AAM could be a novel allogenic injectable product. However, its retention rate poses a concern, as repeated AAM injections may be required in some cases. This study investigated the biological properties and therapeutic value of stored AAM and compared them with those of fresh AAM, in a murine model.

**Methods:**

AAM was manufactured from fresh human abdominoplasty fat. Fresh and stored injectable AAM was prepared within 24 h and 3 months after generation, respectively. Either fresh or stored injectable AAM was injected into the scalp of athymic nude mice (0.2 mL/sample, n = 6 per group). After 8 weeks, graft retention was assessed through weight measurement, and histological analysis was performed, including immunofluorescence staining for CD31 and perilipin.

**Results:**

Retention rate was significantly reduced in the stored compared to the fresh injectable AAM group. Nevertheless, histological analysis revealed comparable inflammatory cell presence, with minimal capsule formation, in both groups. Adipogenesis occurred in both groups, with no significant difference in the blood vessel area (%) between groups.

**Conclusions:**

Although the volume effects of stored AAM for soft tissue reconstruction were limited compared to those of fresh injectable AAM, stored AAM had similar capacity for adipogenesis and angiogenesis. This promising allogeneic injectable holds the potential to serve as an effective “off-the-shelf” alternative for repeated use within a 3-month storage period.

**No Level Assigned:**

This journal requires that authors assign a level of evidence to each submission to which Evidence-Based Medicine rankings are applicable. This excludes Review Articles, Book Reviews, and manuscripts that concern Basic Science, Animal Studies, Cadaver Studies, and Experimental Studies. For a full description of these Evidence-Based Medicine ratings, please refer to the Table of Contents or the online Instructions to Authors https://link.springer.com/journal/00266.

## Introduction

Soft tissue augmentation is a significant requirement in plastic and reconstructive surgery. The number of commercially available soft tissue fillers has increased. Hyaluronic acid-based filler is the most commonly used owing to its excellent immune profile and minimal allergy concerns [1, 2]. However, it does not replicate all characteristics of living tissue and has a volume effect of only up to 1 year; hence, repeated injections are required to maintain the desired outcome. Autologous fat grafting can be considered as a standard for filling and rejuvenation [3]; however, it has a significant drawback in that graft retention is unpredictable. A meta-analysis study involving 1011 patients who underwent facial fat grafting showed fat graft retention ranging from 26 to 83% [4]. Ideal material for soft tissue augmentation should be biocompatible, avoid immune complications, and offer long-term volume maintenance by encouraging new fat formation.

Recently, acellular adipose matrix (AAM) was introduced as a potential soft tissue reconstruction material. This is an extracellular matrix isolated from adipose tissue. Briefly, fat tissue undergoes mechanical, chemical, and biological processes to remove cellular components completely [5]. AAM can potentially act as a scaffold that not only supports stem cells in proliferation and differentiation but also induce adipogenesis and angiogenesis [5, 6]. AAM has minimal immunogenicity [6, 7], serving as an off-the-shelf biocompatible filler in plastic and reconstructive surgery and tissue engineering [8]. Comparable volume effects for injectable AAM and lipoaspirate have been shown previously in a murine model; thus, AAM could be a promising allogenic injectable product [9].

However, the retention rate of AAM is a concern, and repeated injections of AAM may be required in some cases [10]. This study investigated the biological properties and therapeutic value of stored AAM, compared to fresh AAM (i.e., AAM used within 24 hours after generation) in a murine model.

## Patients and Methods

### Adipose Tissue Harvesting and Injectable AAM Processing

The study protocol was approved by the relevant institutional review board. Abdominal adipose tissue samples were sourced from discarded tissues of patients aged 30–40 years undergoing transverse rectus abdominis myocutaneous flap surgery for breast reconstruction. Fat tissue was used to generate AAM using mechanical, chemical, and biological processes, following previously published protocols [9]. Adipose tissue underwent five freeze-thawing cycles and was then centrifuged at 1500×*g* for 10 min [11]. After washing, the fatty portion was discarded, and the tissue was treated with polar solvent extraction using 99.9% isopropanol over 8 h [12]. After washing, the processed tissue was incubated in a solution of 0.05% trypsin, 0.05% EDTA, 10 ng/ml DNAse I (Sigma–Aldrich; Merck KGaA, Darmstadt, Germany), and 10 ng/ml RNAse (Sigma-Aldrich; Merck KGaA) for 2 h, with slow rotation, in an incubator at 37 °C. Finally, the tissue was washed four times with phosphate-buffered saline (PBS) for 30 min each time and subsequently treated with 1% penicillin (Sigma-Aldrich; Merck KGaA) and streptomycin (Sigma-Aldrich; Merck KGaA) for 12 h at 4 °C [13]. It was then dried under ultraviolet light for 4 h at room temperature. To fabricate the “fresh injectable AAM,” the final material was meticulously minced with sharp scissors prior to use within 24 h. AAM was stored at 4 °C in PBS for 3 months prior to use as the “stored injectable AAM” [14]. It was then dried and minced in the same manner as the “fresh injectable AAM”.

### Scanning Electron Microscopy

Fresh and stored AAM samples were analyzed by scanning electron microscopy (SEM) to confirm cellular components and to investigate the matrix architecture. Samples were fixed in 2.5% glutaraldehyde for 24 h at 4 °C. Samples were then moved to a cover glass slide and air-dried at room temperature. The surface morphology of the AAM was observed by SEM (JSM-7401F, JEOL Inc., Akashima, Japan) following coating with platinum at an accelerating voltage of 15 kV.

### Animal Models and Procedures

Eight-week-old athymic nude mice (weight: 20–25 g) were purchased from Koatech (Gyeonggi, South Korea). The mice were housed in a temperature-controlled environment at 24 ± 2 °C, with an artificial 12-h light/dark cycle. All applicable institutional and/or national guidelines for the care and use of animals were followed.

Twelve mice were anesthetized with 3% isoflurane in 100% oxygen at a delivery rate of 5 L/min for anesthesia induction. They were randomly divided into two groups (n = 6 per group): either fresh AAM or stored AAM samples were randomly injected into the mice at the supraperiosteal plane of the skull, using 18-gauge needles (0.2 mL/sample). At postoperative week 8, all mice were anesthetized and the grafts were extirpated. The grafts were evaluated using weight measurement. The graft portion was excised using a scalpel and scissors and subjected to macroscopic photography. Following macroscopic observation of each animal treated with injected fresh and stored AAM, five out of six samples were randomly subjected to histological analysis. All the anesthetized mice were sacrificed at the end of the experiment.

### Histomorphometric Analysis

Five tissue samples in each group were fixed overnight in 4% paraformaldehyde (BD Biosciences, Franklin Lakes, NJ, USA), dehydrated, and embedded in paraffin. Slides of 4-μm thickness were prepared for hematoxylin–eosin staining, to compare the amounts of inflammatory cells, and Masson's trichrome staining, to delineate collagen distribution. Images were observed using a light microscope (Nikon ECLIPSE Ts2, Nikon, Tokyo, Japan). Five random fields were selected and evaluated for inflammation independently by two investigators, based on a semiquantitative scale, ranging from 0 to 5, with 0 = none, 1 = minimal presence (< 20%), 2 = minimal to moderate presence (20–40%), 3 = moderate presence (40–60%), 4 = moderate to extensive presence (60–80%), and 5 = extensive presence (> 80%). The average values were used for analysis.

Unstained slides were deparaffinized with xylene, rehydrated, and boiled in citrate buffer (Agilent Technologies, Santa Clara, CA, USA) for antigen retrieval. Samples were then blocked with 5% goat serum (Jackson ImmunoResearch, West Grove, PA, USA.) for 1 h and incubated overnight at 4 °C with primary antibodies. After washing, samples were incubated with secondary antibodies for 2 h at room temperature. Slides were counterstained with 40,6-diamidino-2-phenylindole to visualize the cell nuclei. The primary antibodies used included rabbit anti-CD31 (PA5-16301; Invitrogen, Waltham, MA, USA) and rabbit anti-perilipin (PA5-72921; Invitrogen). The secondary antibodies used were fluorescein-conjugated goat anti-rabbit. Immunofluorescence-stained images were visualized using a confocal microscope (SP8; Leica, Wetzlar, Germany). Five 500 × 500 μm-sized random fields from each sample were analyzed using ImageJ (Fiji) software (National Institutes of Health, Bethesda, MD, USA) and the average % area values were determined.

Immunofluorescence staining was evaluated in five paraffin block samples from both groups by confocal microscopy. Perilipin staining was employed to detect adipogenesis in the graft materials. For a quantitative evaluation of adipogenesis, perilipin-positive adipocyte counts were measured in five 500 × 500 μm-sized fields.

### Statistical Analysis

The results were analyzed using nonparametric Mann–Whitney U tests because the sample size was small, and data were not normally distributed. R version 4.0.3 (https://www.r-project.org/) was used for statistical analyses, and *p*-values < 0.05 were considered statistically significant.

## Results

### 3D Microstructure of Fresh and Stored AAM

**Fig. 1:**
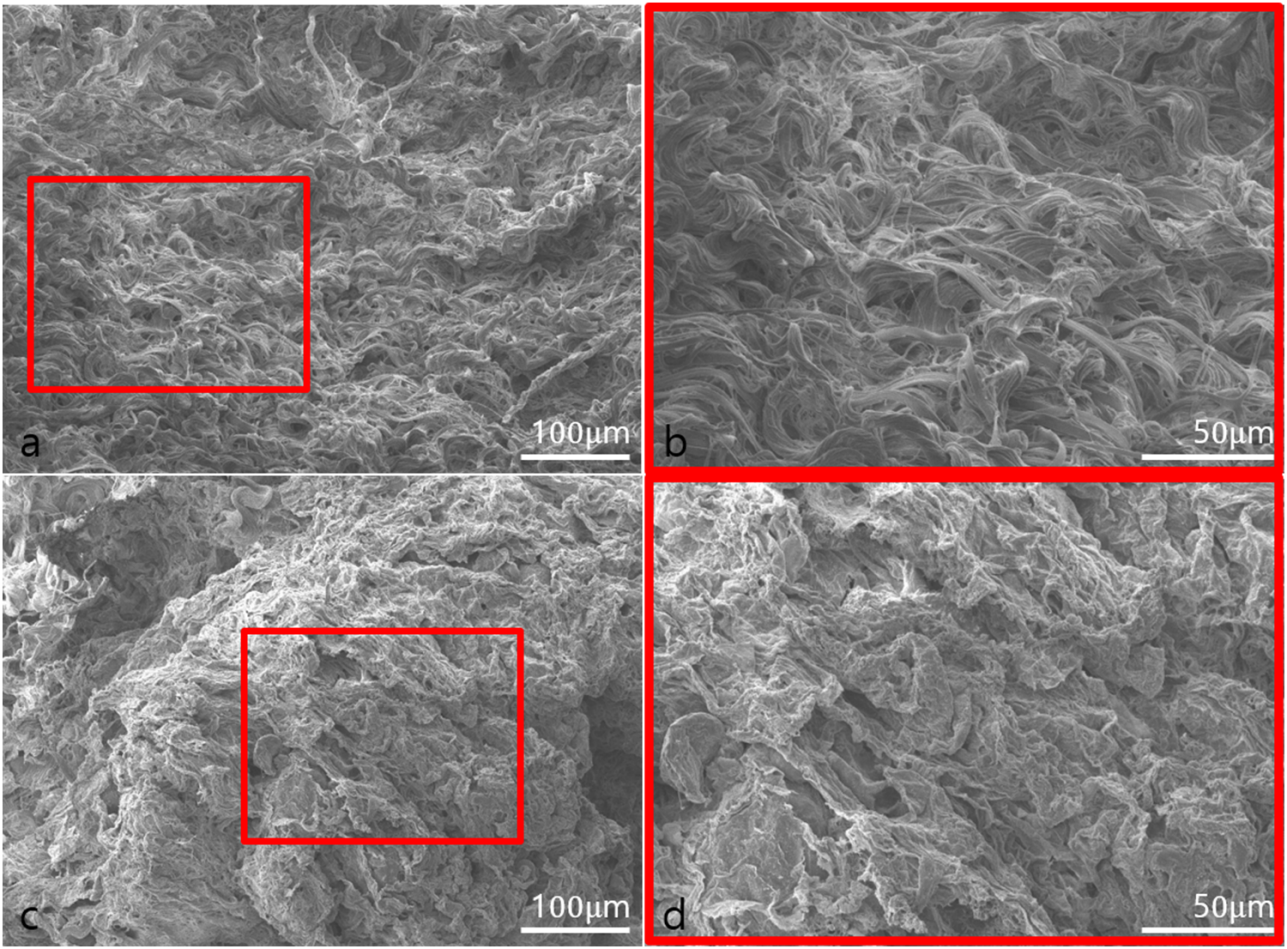
Scanning electron microscopy examination of fresh acellular adipose matrix (AAM) (**a**, **b**) and 3-month-stored AAM (**c**, **d**). Scale bar, 200 × 100 μm (**a**, **c**) and 450 × 50 μm (**b**, **d**)

Microscopic examination of SEM images demonstrated that both fresh and stored AAM exhibited a finely organized, parallel fibrillar structure, devoid of cellular components. By comparing the SEM images of the two preparations, we observed that the fresh AAM consisted of organized nano-fibrous collagen 3D microstructure with more voluminous fibers and micropores. The stored AAM, in contrast, showed a nano-fibrous collagen 3D microstructure with more shrunken and compressed fibers and fewer micropores (Fig. 1).

### Macroscopic Observation of Graft Retention

**Fig. 2:**
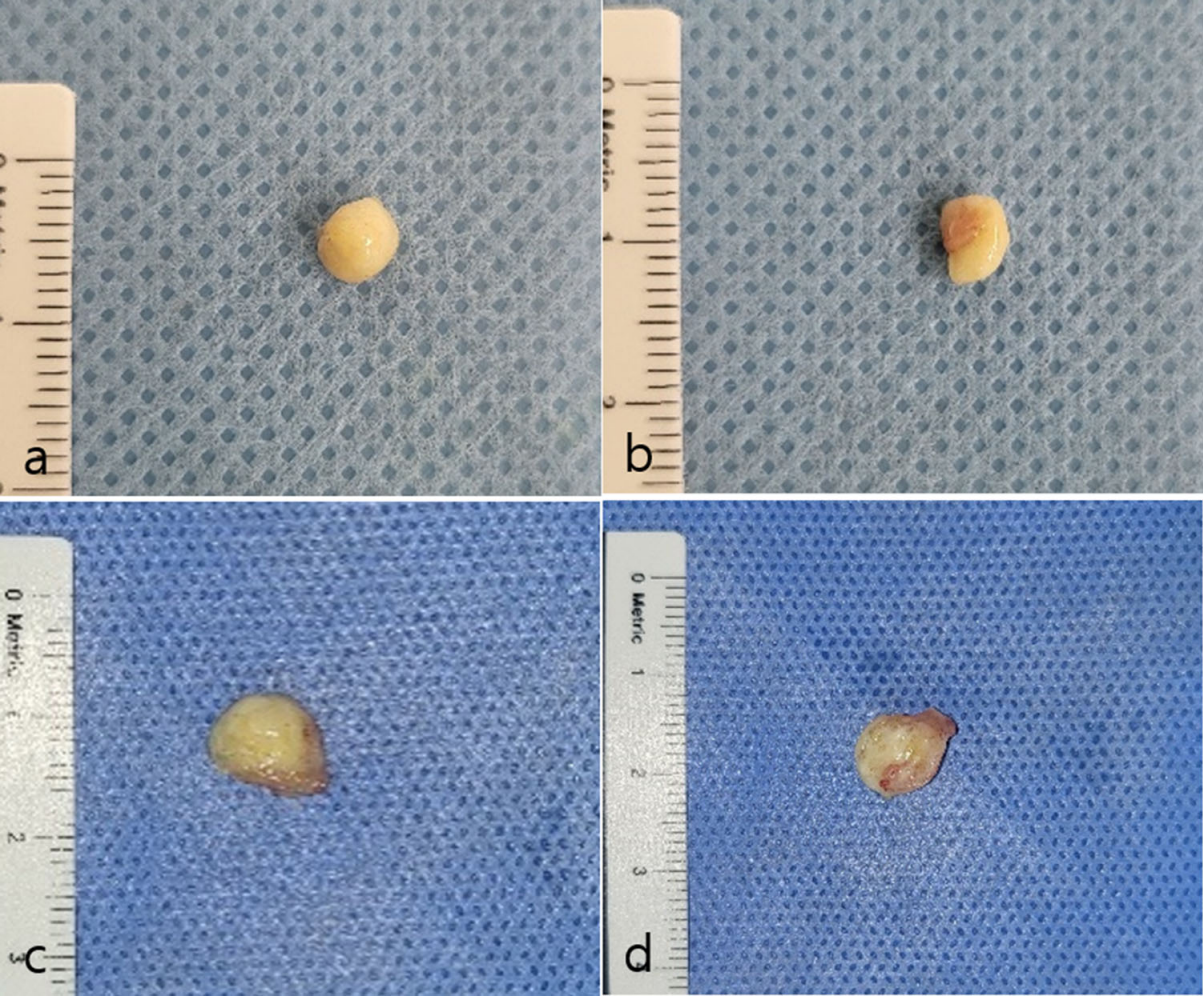
Macroscopic observation of extirpated mass of injected fresh (**a**, **b**) and stored (**c**, **d**) acellular adipose matrix after 8 weeks

Extirpated graft samples in both groups were round and firm with microvasculature present on the surface, which could not be distinguished with the naked eye by two observers. The visible microvasculature highlighted the angiogenic effects of both the fresh and stored AAM (Fig 2).

### Quantitative Analysis of Graft Retention

**Fig. 3:**
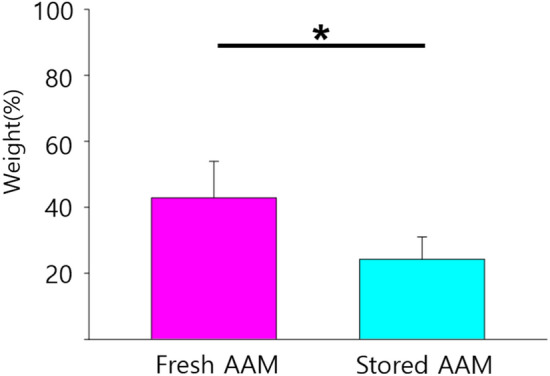
The weight retention (%) of the injected materials are larger in the fresh AAM group than the stored AAM group. The Mann–Whitney U test was used for analysis; data are presented as the mean ± standard deviation; n = 6 per group. AAM, acellular adipose matrix

The mean initial weights from the three measurements of the material present in 0.2 ml of injectable AAM (both fresh and stored) were 0.175 g. The weights of the grafted materials at postoperative week 8 were measured. The retained sample weight (%) of the injected materials was larger in the fresh AAM group than in the stored AAM group (fresh AAM group: median = 44.9%, interquartile range = 35.4–49.1%; stored AAM group: median = 23.4%, interquartile range = 24.0–22.4%, *p* = 0.013, n = 6) (Fig. 3).

### Histological Findings of Graft Retention

**Fig. 4:**
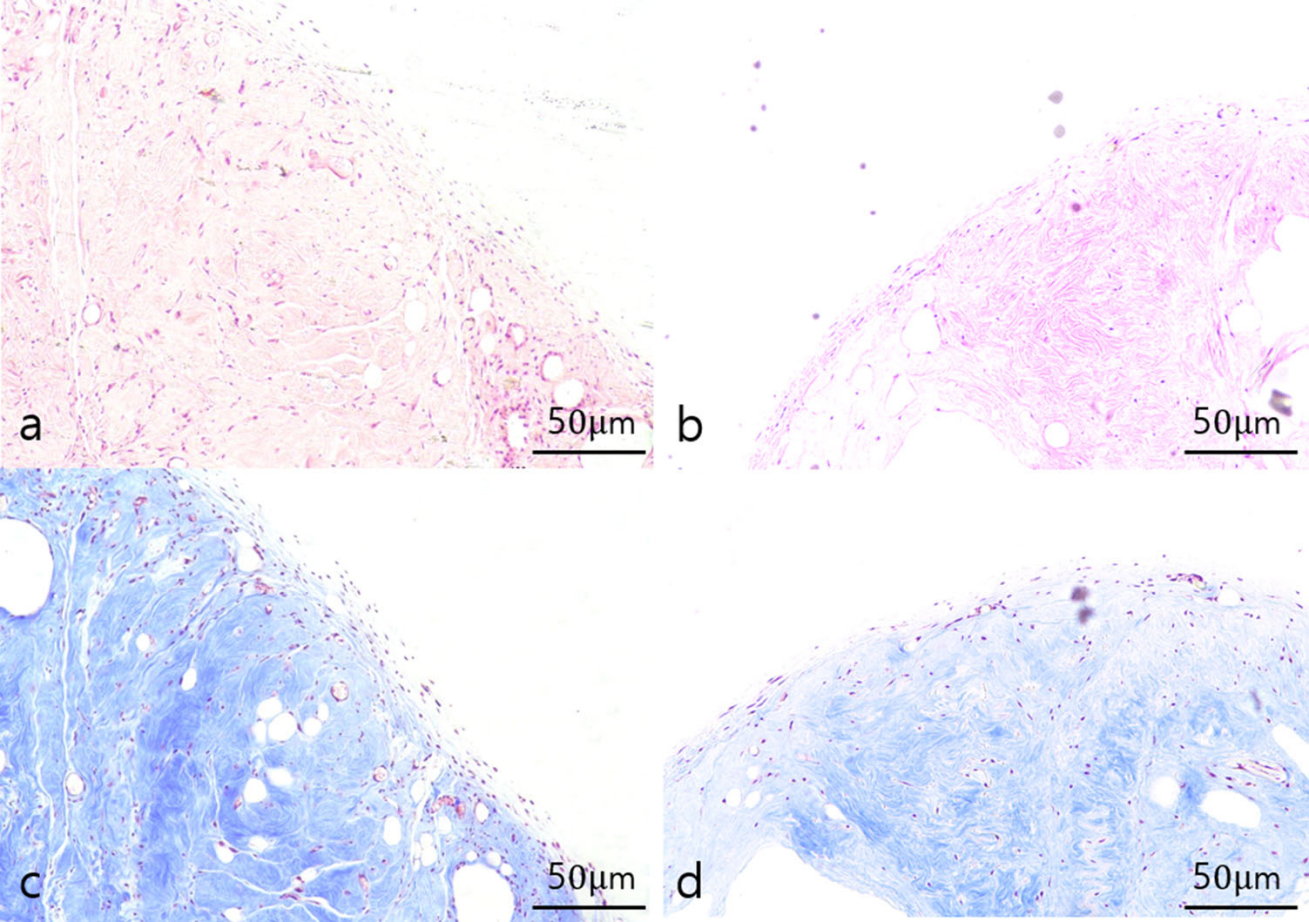
Histological findings of a section of extirpated mass of the fresh (**a**, **c**) and stored (**b**, **d**) AAM group. Inflammatory cell infiltrations are observed in hematoxylin–eosin-stained sections (**a**, **b**) and collagenous fibrous tissue component are observed in Masson’s trichrome-stained sections (**c**, **d**). Magnification 200 ×, scale bar, 50 µm. AAM, acellular adipose matrix

Five randomly selected graft samples from each group were subjected to histological analysis. Inflammatory cell infiltrations are observed in hematoxylin–eosin-stained sections and collagenous fibrous tissue component are observed in Masson’s trichrome-stained sections (Fig. 4). Inflammatory cell infiltration in the grafted materials showed no statistically significant difference between the fresh and stored AAM groups (fresh AAM group: median = 1, interquartile range = 1–2; stored AAM group: median = 1, interquartile range = 1–2, *p* = 0.905, n = 5).

**Fig. 5:**
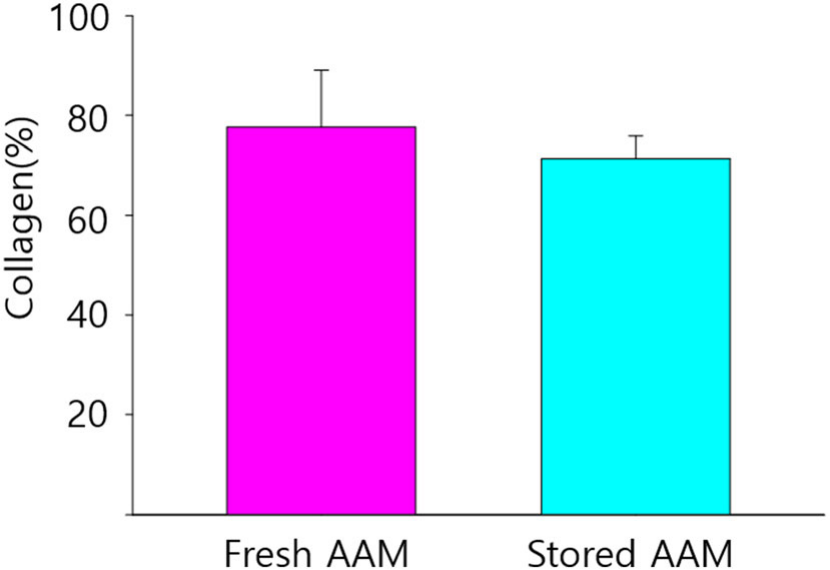
The collagen proportion does not differ significantly between the fresh and stored AAM group (fresh AAM group: median = 84.7%, interquartile range = 71.7–85.4%; stored AAM group: median = 73.1%, interquartile range = 71.2–74.4%, *p* = 0.403, n = 5). AAM, acellular adipose matrix

For a quantitative evaluation of collagen proportion, %area values, representing the collagen %area, were measured in five 500 × 500 μm-sized fields. We found no significant difference in collagen proportion between the fresh and stored AAM groups (fresh AAM group: median = 84.7%, interquartile range = 71.7–85.4%; stored AAM group: median = 73.1%, interquartile range = 71.2–74.4%, *p* = 0.403, n = 5) (Fig. 5).

### Analysis of Adipogenesis and Angiogenesis

**Fig. 6:**
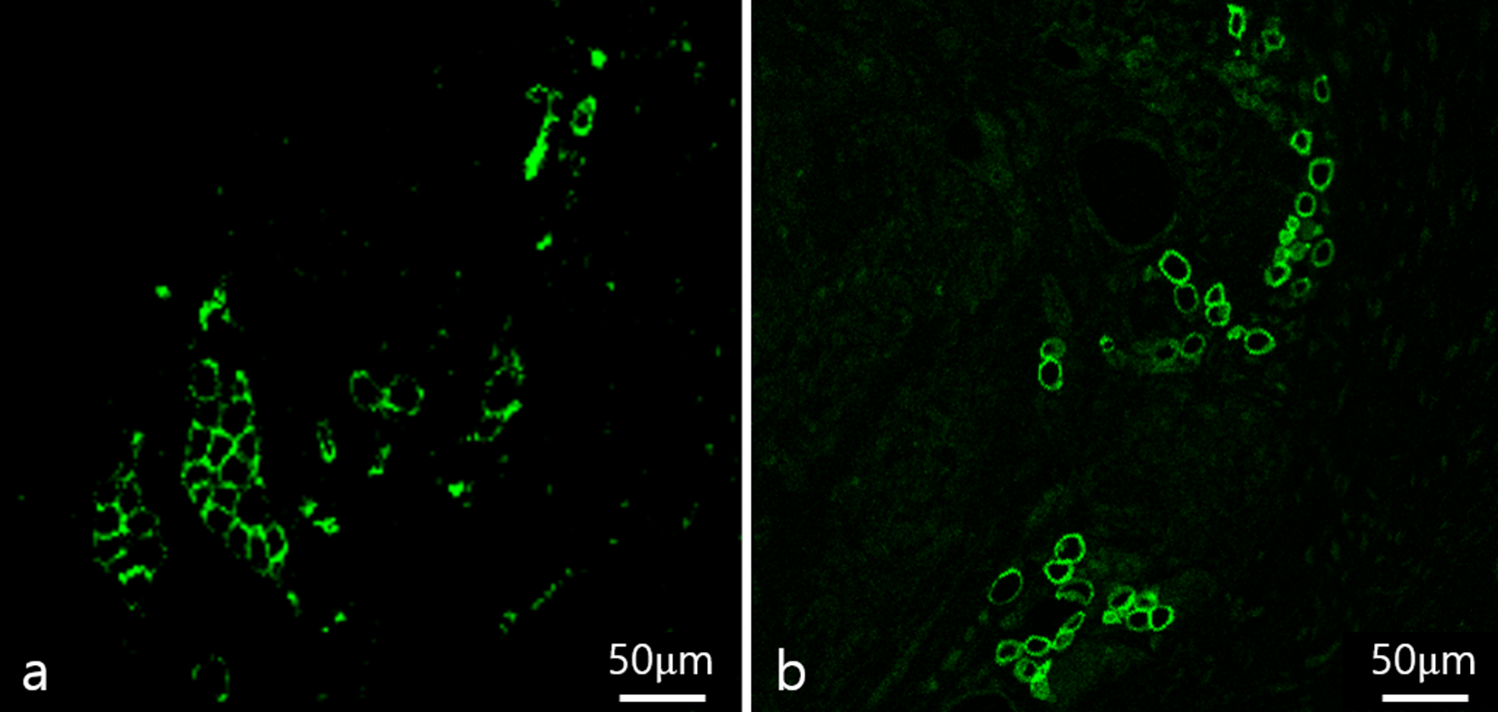
Immunofluorescent staining of adipocytes of a section of fresh (**a**) and stored (**b**) AAM group. The green signals refer to perilipin (+) adipocytes, which imply adipogenesis. Scale bar, 50 µm. AAM, acellular adipose matrix

**Fig. 7:**
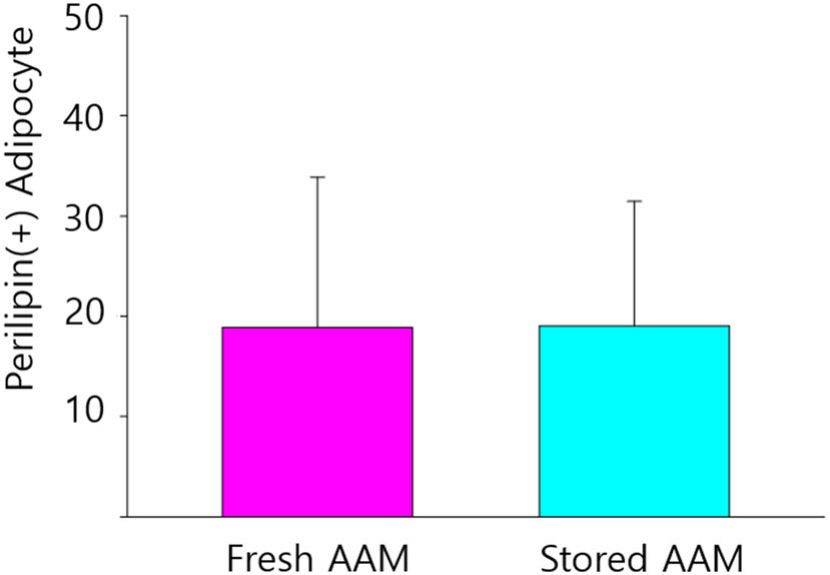
The perilipin (+) adipocyte count in the fresh and stored AAM group are not significantly different. The Mann–Whitney U test was used for analysis; data are presented as the mean ± standard deviation; n = 5 per group. AAM, acellular adipose matrix

We found no significant difference in perilipin-positive adipocyte number between the fresh and stored AAM groups (fresh AAM group: median = 17.6, interquartile range = 4.4–31.6; stored AAM group: median = 14.8, interquartile range = 12.4–17.2, *p* = 0.999, n = 5) (Figs. 6 and 7).

**Fig. 8:**
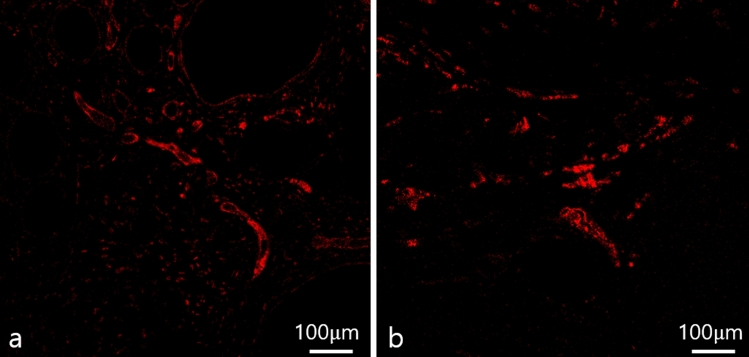
Immunofluorescent staining of endothelial cells of a section of fresh (**a**) and stored (**b**) AAM group. The red signals refer to CD 31 (+) cells, which imply angiogenesis. Scale bar, 100 µm. AAM, acellular adipose matrix

**Fig. 9:**
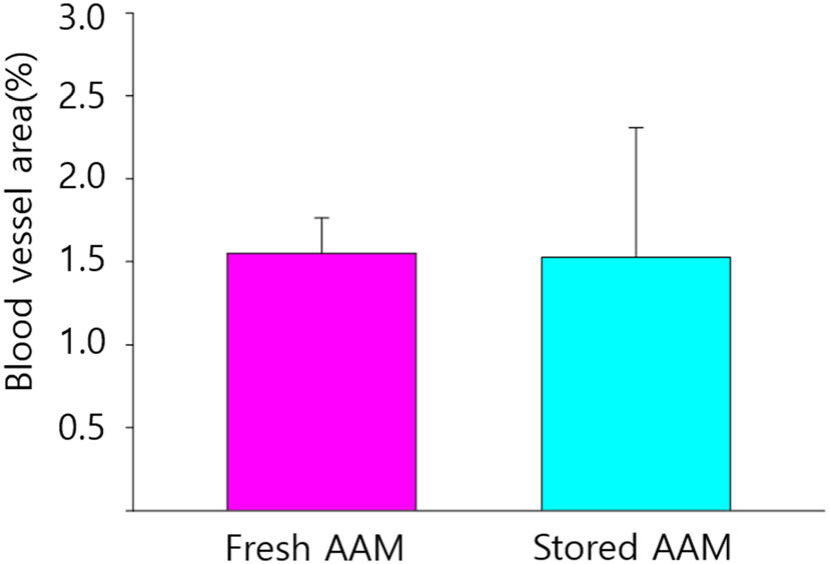
The blood vessel area (%) in the fresh and stored AAM group are not significantly different. The Mann–Whitney U test was used for analysis; data are presented as the mean ± standard deviation; n = 5 per group. AAM, acellular adipose matrix

CD31 staining was employed to identify angiogenesis, a marker of endothelial cells in blood vessels. Many perilipin-positive cells were observed in both fresh and stored AAM groups (Fig. 8). For a quantitative evaluation of angiogenesis, the %area values, representing the blood vessel area (%) in five 500 × 500 μm-sized fields were randomly determined. CD31-positive staining did not differ between the fresh and stored AAM groups (fresh AAM group: median = 1.61%, interquartile range = 1.42–1.72%; stored AAM group: median = 1.24%, interquartile range = 1.13–1.35%, *p* = 0.2101, n = 5) (Fig. 9).

## Discussion

Although injectable AAM has shown effectiveness comparable to conventional fat grafting, the issue of effectiveness depending on storage period had to be identified [9]. To increase the utility value of a product as a “off-the-shelf” merchandise, its effectiveness should be maintained as much as possible throughout the storage period. Therefore, additional research was planned considering that the product should be utilizable during its storage period. Here, we investigated the biological properties and therapeutic value of stored AAM, as compared to fresh AAM (i.e., AAM used within 24 h after generation) in a murine model. The retention rate was significantly reduced in the stored compared to the fresh injectable AAM group. Nevertheless, histological analysis revealed comparable inflammatory cell presence, adipogenesis, and angiogenesis, with minimal capsule formation, in both the fresh and stored injectable groups.

A previous study showed that the volume effect of AAM is similar to that of clinically well-known conventional fat grafting [9]. Furthermore, AAM has potential clinical benefit because of its convenience. AAM can be used more easily in patients than lipoaspirate, which should be harvested from donor sites of the patients prior to injection procedures [15]. This material could reduce not only time but also physical and psychological strain of the patients due to its unique advantages and convenience [8], and could avoid donor site morbidity. Thus, although using AAM as an “off-the-shelf” product may incur a financial burden, it remains an attractive and competitive material from a cost-effectivity point of view.

In various previous basic studies related to AAM material, the prepared AAM was usually stored in a 1% penicillin and streptomycin solution at 4 °C [16–20]. Although the impact of the storage method has not been accurately analyzed, we followed this previous storage method as an up-to-date reference.

In the in vitro study, the macroscopic and microscopic structure of the product was preserved after storage for 3 months, although there was a difference in the SEM microstructure. The density of the fresh and stored material remained similar. The in vivo study revealed that the volume effect was reduced in the stored AAM group; however, we showed, both qualitatively and quantitatively, that the unique properties of AAM, i.e., adipogenesis and angiogenesis, were preserved. Therefore, it can be assumed that the volume effect partially decreases depending on the storage period due to factors other than these two main characteristics. Additional research will be needed to confirm the mechanism.

Moreover, this study revealed that unlike conventional autologous fat, the effectiveness of AAM is limited if it is stored for a certain period of time after production. Nevertheless, although it is better to use the product as soon as possible after production, it can be stored for a while. This could increase the ease of distribution of this material that can be manufactured from discarded human fat. Furthermore, this could be a clinical merit as compared to conventional fat grafting, as an individual patient could then store the remaining product after the first use and could then repeat the procedure after 3 months when partial absorption occurred or to increase the volume in soft tissue reconstruction, and repeatedly refine the contouring process. This would therefore increase cost-effectiveness.

This study had a few limitations. First, as it was not a clinical study, the same product may lead to different results in clinical settings. Second, only a few mice were included in each group, which might have led to random errors or a low statistical power. Third, unintentional performance bias might have occurred while injecting AAM or extirpating the grafted mass sample in the two groups, as this was a labelled-group experiment. Additionally, serial evaluations, such as 1-month, 6-month, or longer follow-up evaluations were not performed. Finally, there was no precise evaluation of safety and complications according to the storage period.

## Conclusion

We showed that 3-month-stored injectable AAM demonstrated a limited volume effect in comparison to that of fresh injectable AAM in soft tissue reconstruction. However, the ability of adipogenesis and angiogenesis of the fresh and stored AAM was similarly preserved. This promising allogeneic injectable holds the potential to serve as an effective “off-the-shelf” alternative for repeated use for soft tissue reconstruction within a 3-month storage period. Further studies are needed to assess changes in AAM characteristics and utility over time and to determine the safe storage period for this material.
